# TPT: a compact CNN-transformer encoder for efficient microbial small protein modeling

**DOI:** 10.3389/fmicb.2026.1839420

**Published:** 2026-08-03

**Authors:** Fang Sheng, Junhe Zhang, Chengkai Zhu

**Affiliations:** 1 Institute of Science and Technology for Brain-Inspired Intelligence, Fudan University, Shanghai, China; 2 University of Toronto, Toronto, ON, Canada; 3 Department of Thoracic Surgery, Zhongshan Hospital, Fudan University, Shanghai, China

**Keywords:** deep learning, microproteins, peptide prediction, protein language model, smORFs

## Abstract

**Introduction:**

Microbial small proteins, encoded by small open reading frames (smORFs), play essential roles in antimicrobial activity, metabolic regulation, and signaling pathways. However, their short length and rapid evolutionary rate present significant challenges for computational modeling.

**Methods:**

We introduce TinyProteinTransformer (TPT), a lightweight CNN-Transformer hybrid encoder pretrained on the Global Microbial smORF Catalog (GMSC; >280 million smORF families). TPT integrates multi-scale convolutional filters to capture local sequence motifs with Transformer layers for broader contextual modeling, and is trained jointly with masked language modeling and contrastive learning to capture both residue-level and sequence-level representations. We evaluated TPT on six downstream peptide/protein classification benchmarks spanning antimicrobial peptides (AMP), toxic peptides (TOX), bacteriocins (BCN), anti-CRISPR proteins (Acr), quorum-sensing peptides (QSP), and cell-penetrating peptides (CPP) using frozen-encoder linear probing.

**Results:**

On the AMP and TOX tasks, TPT (103 M parameters) matched ESM2-150 M in predictive performance (AUC 0.930 vs. 0.928 for AMP; 0.930 vs. 0.925 for TOX) while achieving 4.3-fold faster inference. Across the evaluated benchmarks, TPT showed competitive performance compared with larger protein language models. Ablation experiments identified the contrastive objective as an important contributor to representation quality: its removal reduced AUC across all six downstream tasks and produced performance patterns consistent with MLM-only pretraining.

**Conclusion:**

These results suggest that, within the evaluated benchmarks, effective smORF representation may benefit from pretraining objectives and inductive biases tailored to short, rapidly evolving sequences, rather than from model scale alone. TPT therefore provides a compact and computationally efficient encoder with competitive performance for microbial peptide analysis.

## 1 Introduction

Small proteins, also referred to as peptides encoded by small open reading frames (smORFs), are generally considered to consist of 100 amino acids or fewer. These peptides play crucial biological roles in antimicrobial activity, metabolic regulation, intercellular signaling, and adaptation to environmental stressors (Duval and Cossart, 2017; Sberro et al., 2019; Storz et al., 2014). In eukaryotes, smORF-encoded small proteins have attracted growing attention for their roles in diverse biological processes and disease mechanisms, including embryogenesis (Pauli et al., 2014; Savard et al., 2006; Hao et al., 2025), stress responses (Andreev et al., 2015; Khitun et al., 2019), muscle development (Anderson et al., 2015; Bi et al., 2017; Matsumoto et al., 2017), immune and inflammatory responses (Bhatta et al., 2020; Jackson et al., 2018; Nichols et al., 2024), and tumorigenesis (Huang et al., 2021; Lyu et al., 2023; Zhang et al., 2025). Recent advances in high-throughput genome sequencing and ribosome profiling have demonstrated that smORFs are far more abundant and functionally diverse than previously recognized (Ingolia et al., 2009; Miravet-Verde et al., 2019; Weaver et al., 2019). However, their short length, rapid evolutionary turnover, and sparse domain organization pose unique challenges for computational characterization (Couso and Patraquim, 2017).

Earlier peptide prediction methods, such as AMPlify (Li et al., 2022) and AMPScannerV2 (Veltri et al., 2018), mainly relied on task-specific supervised learning frameworks for antimicrobial peptide prediction. Although effective within individual tasks, these non-pretrained models are typically trained on limited labeled datasets and may have reduced generalizability across diverse peptide functions. Protein language models (PLMs) have proven highly effective in learning representations from large unlabeled protein sequence corpora. In particular, models like ESM (Lin et al., 2023), ProtTrans (ProtBERT, ProtT5) (Elnaggar et al., 2021), and MSA Transformer (Rao et al., 2021) have demonstrated strong performance in structure prediction, mutation effect prediction, and functional annotation. However, these models are predominantly trained on databases such as UniRef50 and UniRef90 (Suzek et al., 2007), which largely consist of long, evolutionarily conserved proteins. Consequently, their architectural and pretraining assumptions are often oriented toward long-range attention, global structural dependencies, and evolutionary alignment signals, which may not be optimally suited to the highly local, motif-driven signatures characteristic of smORF-encoded small proteins. More recently, models like MCWS-Transformers (Ranjan et al., 2023) have further highlighted the importance of modeling local sequence contexts in protein representation learning. These developments suggest that compact architectures with enhanced local motif extraction may be particularly useful for microbial smORF-encoded small protein modeling.

**Figure 1:**
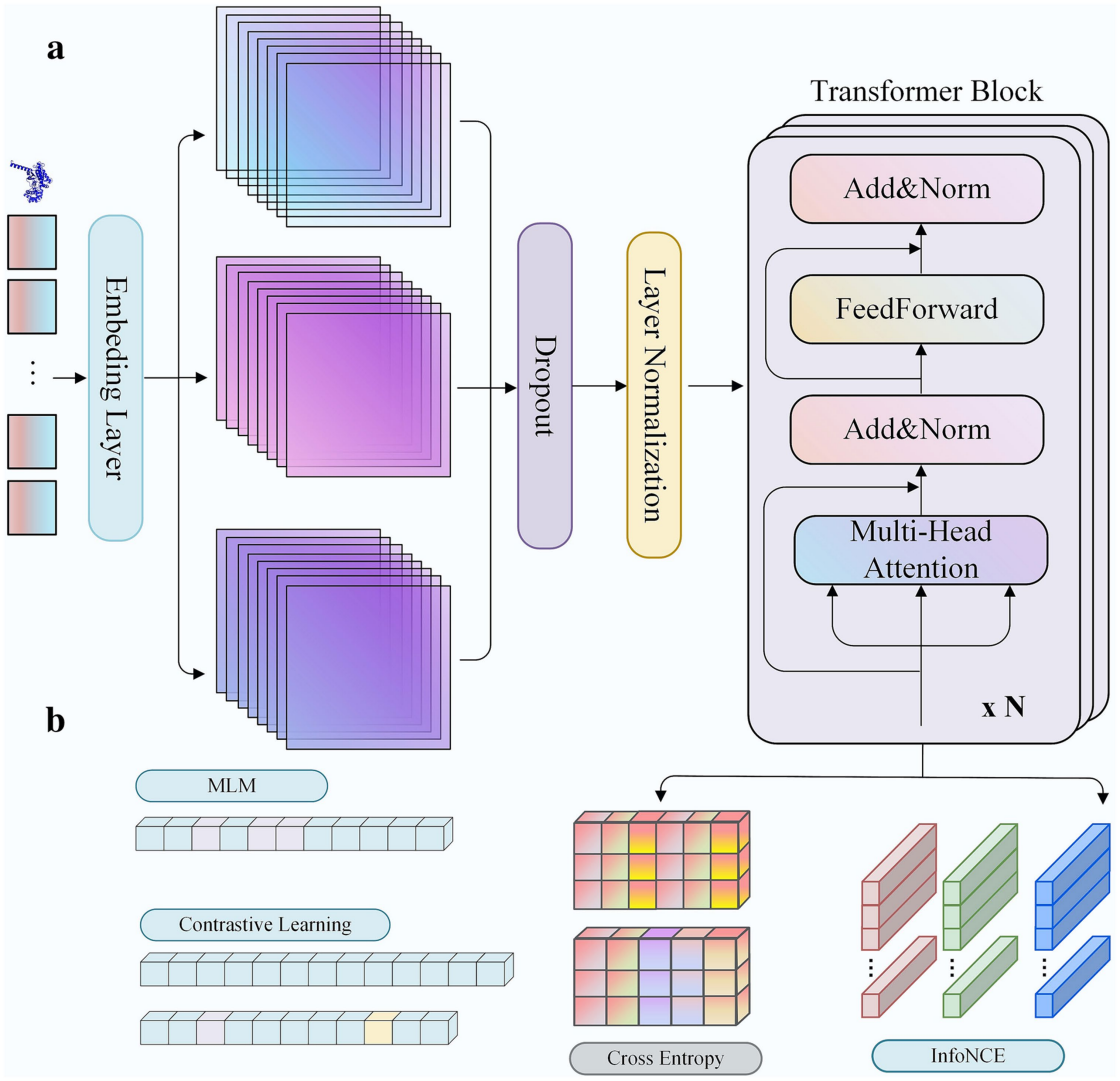
Architecture of TinyProteinTransformer and its self-supervised training objectives. **(a)** Model architecture. The TinyProteinTransformer (TPT) combines multi-scale convolutional filters with deep Transformer encoder blocks to model both local motifs and global contextual features in small proteins. Input sequences (≤128 aa) are embedded with learned token and positional encodings, passed through four parallel 1D convolutions (kernel sizes 3/5/7/9), and fused via residual projection. The resulting features are processed by stacked Transformer layers, and a learned attention-pooling module aggregates residue representations into a fixed-length embedding. **(b)** Pretraining objectives. TPT is trained with two complementary self-supervised tasks: masked language modeling (MLM), where 15% of residues are masked and predicted using cross-entropy loss; and contrastive learning, where two augmented views of each peptide are encoded and optimized with an InfoNCE loss to bring paired representations together while separating negatives. The combined objectives shape both token-level and sequence-level representations.

In this study, we introduce TinyProteinTransformer (TPT), a lightweight CNN–Transformer hybrid encoder specifically designed for microbial smORF-encoded small proteins (Figure 1). The main contributions of this study are as follows: (1) We propose a compact CNN-Transformer architecture that combines multi-scale convolutional filters with Transformer-based contextual modeling to capture local motifs and sequence-level dependencies in short peptides. (2) We develop a smORF-adapted pretraining strategy on the GMSC dataset by integrating masked language modeling with contrastive learning. (3) We evaluate TPT across multiple downstream peptide/protein classification tasks and further examine its key components through ablation, hidden-dimension, representation, and efficiency analyses. Through this combination of architecture and training strategy, TPT achieves competitive performance while maintaining high computational efficiency across the evaluated downstream tasks, compared with established protein language models such as the ESM2 and Prot series.

## 2 Materials and methods

### 2.1 Pretraining dataset

TinyProteinTransformer (TPT) was pretrained on representative sequences from the Global Microbial smORF Catalog (GMSC) reported by Duan et al. (2024). The GMSC was constructed from 63,410 assembled metagenomes and 87,920 isolate microbial genomes from 75 countries, and contains 287,926,875 non-redundant smORF families clustered at 90% amino acid identity and 90% coverage (GMSC10.90). In this study, we used the officially released version of GMSC10.90 without additional processing. All sequences were truncated to a maximum length of 128 residues.

To assess potential data leakage between the GMSC pretraining corpus and the downstream benchmarks, we performed a two-level sequence similarity analysis across all six downstream datasets. First, exact string matching was applied by streaming all pretraining sequences and checking against the complete set of benchmark sequences. Second, near-identity search was conducted using MMseqs2 easy-search (Steinegger and Söding, 2018) at 90% sequence identity and 80% minimum coverage (−-min-seq-id 0.90 -c 0.80 --cov-mode 0), corresponding to the redundancy threshold used in the original GMSC construction. Exact match rates and near-identity overlap rates per dataset are reported in Supplementary Table S1.

### 2.2 Model architecture

The model employs pre-layer normalization (norm_first = True) for training stability; CNN kernel sizes of 3, 5, 7, and 9 were chosen to span typical functional motif lengths observed in short proteins; dropout of 0.05 was set conservatively given the large pretraining corpus; and the contrastive temperature *τ* = 0.1 and weighting coefficient *λ* = 0.05 follow standard InfoNCE practice, with λ set low to ensure MLM serves as the dominant training signal. Key architectural and training hyperparameters are listed in Supplementary Table S2.

#### 2.2.1 Input representation

For each amino acid sequence input to the model with a length less than 128, it is tokenized using a custom vocabulary: 20 canonical amino acids, ambiguity symbols, masking token, and a padding token. Tokens are then mapped to a 640-dimensional embedding via a learned lookup table (Equation 1):


$$e_{i}=E(x_{i})\in {\mathbb{R}}^{640},x=(x_{1},x_{2},\ldots ,x_{L}),L\leq 128$$ (1)

To encode residue positions, a trainable positional embedding is used (Equation 2):


$$p_{i}=P(i)\in {\mathbb{R}}^{640}$$ (2)

The positional embedding and token embeddings are summed to each other, and sequences shorter than 128 residues are padded (Equation 3):


$$\begin{array}{c} H_{0}(i)=e_{i}+p_{i} \end{array}\;\;$$ (3)

#### 2.2.2 Multi-scale convolutional motif encoder

A parallel multi-scale 1D convolutional module captures local sequence motifs complementary to global Transformer self-attention, with kernel sizes 3, 5, 7, and 9 spanning the range of typical short-protein functional motif lengths. The number of output channels per branch is 256, and the activation function is ReLU. For each kernel size K (Equation 4–5):


$$\begin{array}{c} C_{k}=\text{ReLU}(\text{Conv}1D_{k}(H_{0})),\text{where}\;C_{k}\in {\mathbb{R}}^{L\times 256} \end{array}$$ (4)

$$\begin{array}{c} H_{1}=\text{LayerNorm}(H_{0}+W_{\text{proj}}[\begin{array}{l} C_{3};C_{5};\\ C_{7};C_{9} \end{array}]+b_{\text{proj}})\in {\mathbb{R}}^{L\times 640} \end{array}$$ (5)

#### 2.2.3 Transformer layers

Global sequence semantics are modeled using a 20-layer Transformer encoder (PyTorch nn. TransformerEncoder), with layer normalization applied in advance to improve stability. Stacking 20 such layers yields the final contextual representation (Equation 6):


$$\begin{array}{c} H_{\mathrm{enc}}=\text{TransformerEncoder}(H_{1})\in {\mathbb{R}}^{L\times 640} \end{array}$$ (6)

#### 2.2.4 Attention-based global pooling

To condense the residue-level representations into a fixed-length embedding suitable for contrastive learning and downstream tasks, we introduced a learned attention pooling operator. Each position i is assumed to have a scalar importance score (Equation 7):


$$\begin{array}{c} s_{i}=w^{⊤}h_{i},w\in {\mathbb{R}}^{640} \end{array}$$ (7)

These scores are converted into normalized attention weights through softmax (Equation 8):


$$\begin{array}{c} \alpha_{i}=\frac{\exp(s_{i})}{\sum_{j=1}^{L}\;\exp(s_{j})} \end{array}$$ (8)

The final sequence embedding is the weighted average (Equation 9):


$$\begin{array}{c} v=\sum_{i=1}^{L}\;\alpha_{i}h_{i}\in {\mathbb{R}}^{640} \end{array}$$ (9)

This allows the encoder to emphasize residue encoding functional motifs while suppressing padding and low-information positions.

#### 2.2.5 Gated attention

To improve the selectivity of global context modeling, we incorporate a head-specific gated self-attention into each Transformer encoder layer (Vaswani et al., 2017; Qiu et al., 2025). The attention output (Equation 10):


$$\begin{array}{c} Y\in {\mathbb{R}}^{L\times d} \end{array}$$ (10)

It is modulated by a sigmoid gate computed from the input hidden states (Equation 11):


$$\begin{array}{c} G=\sigma (HW_{g}),\mathrm{Wg}\in {\mathbb{R}}^{d\times H} \end{array}$$ (11)

Where H denotes the number of attention heads. The attention output is reshaped into a head-wise form, multiplied by the gating scores, and then merged. This gating is applied head-wise and was tested as an optional variant (TPT_gated) in downstream tasks; the base model uses standard multi-head attention. We hypothesized that head-specific gating might improve selectivity on tasks dominated by localized functional motifs.

### 2.3 Self-supervised Pretraining

We pre-trained TinyProteinTransformer in a purely self-supervised fashion using masked language modeling at the residue level and contrastive representation learning at the sequence level.

#### 2.3.1 Masked language modeling

To learn residue-level semantics and short-range patterns, we adopted a Bert-style masked language modeling target for the small protein sequence (Devlin et al., 2019). Each input peptide was tokenized and filled to a fixed length of 128 residues. The token set includes 20 typical amino acids, the MASK token, and the PAD token. During the loss calculation process, the filling position is ignored.

To construct the mask prediction target, we combine a span mask and a per-token mask.

For each sequence, we aim to choose 15% of the residues as the targets for masking. The model performs span masking with a 30% probability, where short consecutive fragments (2–5 residues) are removed and replaced with <MASK> tokens. This encourages the model to learn how to reconstruct local motifs and short functional patterns. Under the remaining 70% probability, token-masking positions are selected at the same 15% masking rate.

During the masking period, the replaced positions are recorded, and all unmasked positions are assigned an ignore tag to prevent any loss. The obtained mask sequence is then passed through the encoder to obtain the representations. The linear prediction head projects each hidden state back into the vocabulary space, generating a distribution over all possible amino acid tokens for each position.

The MLM loss was computed using standard cross-entropy (Equation 12):


$$\begin{array}{c} {ℒ}_{\mathrm{MLM}}=\frac{1}{|ℳ|}\sum_{i\in ℳ}\;\mathrm{CE}({\hat{z}}_{i},x_{i}) \end{array}$$ (12)

Where ℳ denotes the set of masked residues, $x_{i}$ is the original amino acid at position *i*, and ${\hat{z}}_{i}$ is the model’s predicted distribution.

The encoder learns to infer the identity of masked residues from their surroundings, thereby being forced to capture motif patterns and short-range dependencies.

#### 2.3.2 Contrastive learning

To better learn the complete features of peptides, we added sequence-level supervision to the training process.

The first type of augmentation is random deletion. A small continuous segment of amino acids (usually 2 to 5) in the peptide will be deleted with a 30% probability. This operation imitates small, naturally occurring truncations in peptides.

The second transformation is conservative substitution, which randomly selects an amino acid position with a 15% probability and replaces it with another amino acid with similar chemical properties. For instance, hydrophobic amino acids (such as I, V, L), aromatic ones (F, W, Y), or positively charged ones (K, R, H). It imitates common mutations in nature that do not alter their core functions or structures. After processing, each sequence is padded to 128 tokens, so that each original peptide chain corresponds to two input data points for contrastive learning.

Following InfoNCE (van den Oord et al., 2018), L2-normalized embeddings from the two augmented views are compared using dot-product similarity at temperature *τ* = 0.1, with same-sequence pairs as positives and all other in-batch pairs as negatives (Equation 13):


$$\begin{array}{c} {ℒ}_{\mathrm{con}}=-\frac{1}{B}\sum_{i=1}^{B}\;\log\frac{\exp(\mathrm{sim}(i_{\left(1\right)}^{z},i_{\left(2\right)}^{z})/\tau )}{\sum_{j=1}^{B}\;\exp(\mathrm{sim}(i_{\left(1\right)}^{z},j_{\left(2\right)}^{z})/\tau )} \end{array}$$ (13)

In each training batch, there are both mask sequences for training the mask language model (MLM) and pairs of augmented sequences for contrastive learning. We train the entire model in an end-to-end manner. The final training loss is the weighted sum of these two losses: the total loss is equal to the MLM loss plus *λ* multiplied by the contrastive loss (where λ takes a value of 0.05). This setting makes the MLM loss the primary training signal while allowing the contrastive loss to serve as a regularization term, helping optimize the model’s global embedding space.

These two training objectives share the encoder parameters and the attention pooling module. Therefore, the model can obtain complementary supervisory information.

#### 2.3.3 Pretraining procedure

All pre-training experiments were conducted using the representative peptide sequence of GMSC10.90. The end-to-end optimization of the TinyProteinTransformer encoder was performed using the AdamW optimizer (Loshchilov and Hutter, 2019) with a learning rate of 2e-5. We used the autocast and GradScaler modules of PyTorch (Paszke et al., 2019) for mixed-precision training to enhance memory efficiency and throughput on NVIDIA GPUs (two NVIDIA RTX 5090 s). When multiple GPUs are available, the model is wrapped in a data-parallel manner, synchronizing gradients across devices at each optimization step.

In each training step, the model simultaneously generates MLM token predictions and embeds them into two augmented sequences. The total loss is calculated as the weighted sum of the MLM and InfoNCE losses (weight = 0.05).

**Figure 2:**
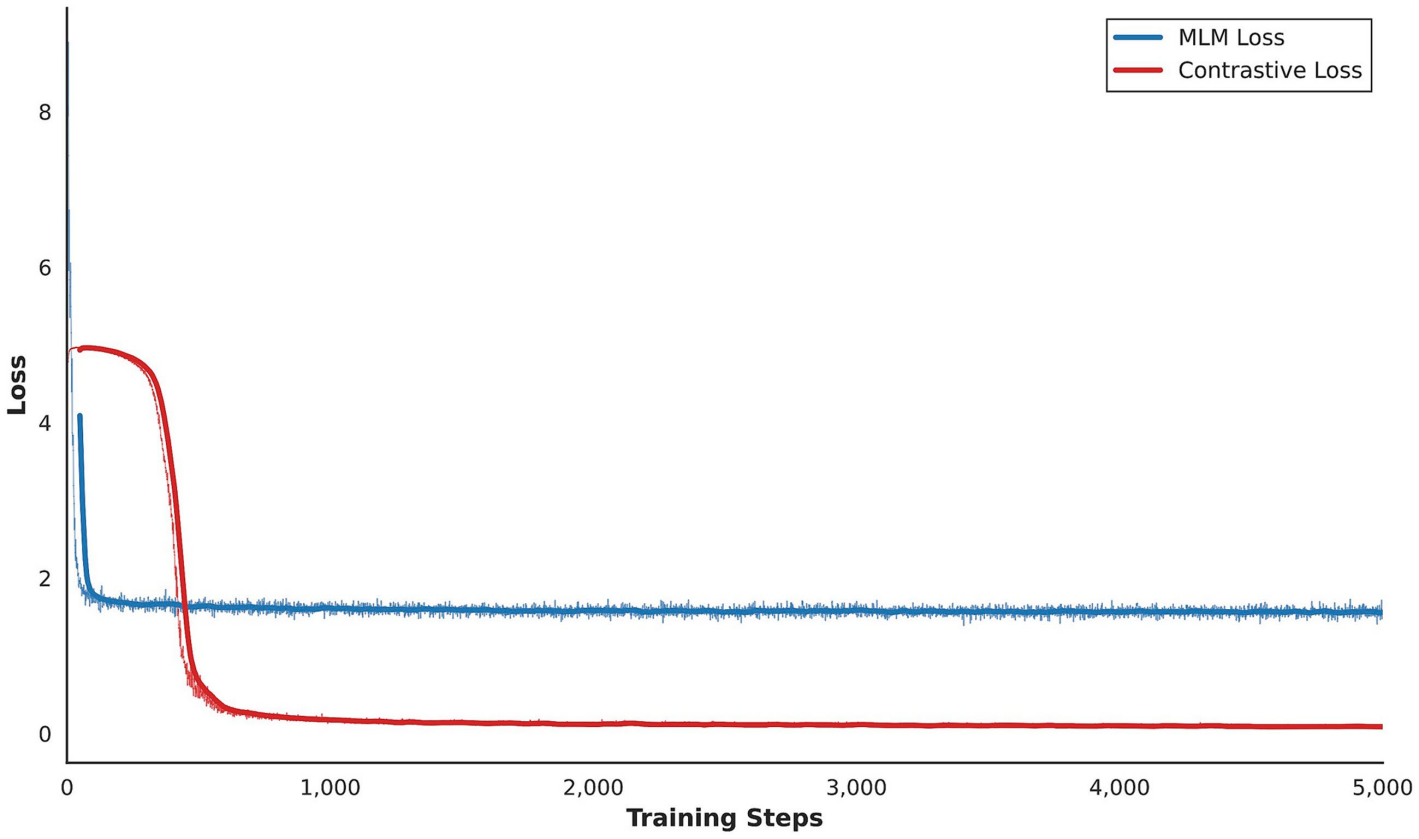
Retraining loss dynamics of TinyProteinTransformer on the GMSC90 dataset. Training curves for masked language modeling (MLM) and contrastive loss. Both losses exhibit a rapid initial decrease followed by stabilization (note the axis scaling in the plateau region).

A global random seed of 42 was set for reproducibility. No validation split was used during self-supervised pre-training; convergence was monitored via training loss curves (Figure 2). The best model checkpoint was selected based on the lowest average loss across epochs.

Three epochs of pre-training were conducted on the entire GMSC dataset. Due to the large number of sequences and mixed self-supervised targets in each epoch, this was sufficient to enable the model to reach a stable loss platform. The resulting pre-trained model serves as the initialization for all downstream classification and representation learning evaluations.

### 2.4 Downstream evaluation

We performed supervised binary classification on six benchmark datasets spanning functionally distinct classes of microbial and therapeutic peptides: the AMPlify antimicrobial peptide (AMP) dataset, the ToxMSRC toxic peptide (TOX) dataset, the BaPreS bacteriocin (BCN) dataset, the PaCRISPR anti-CRISPR (Acr) dataset, the Quorumpeps quorum sensing peptide (QSP) dataset, and the pLM4CPPs cell-penetrating peptide (CPP) dataset.

For antimicrobial peptide prediction, we used the final non-redundant benchmark dataset released by the AMPlify framework (Li et al., 2022). This dataset contains experimentally verified AMP sequences derived from public peptide databases, including APD3 and DADP, as well as non-AMP sequences selected from UniProtKB/Swiss-Prot. Redundancy reduction was performed in the original AMPlify study using CD-HIT (Li and Godzik, 2006) at 90% AAI. We used the published benchmark without additional processing, comprising 4,173 AMP sequences and 4,173 non-AMP sequences.

For toxicity prediction, we used the benchmark dataset established in the ToxMSRC framework (Zhang et al., 2025). Toxic peptides were collected from CSM-Toxin (Morozov et al., 2023), ToxinPred2, and ATSE (Wei et al., 2021). Non-toxic sequences were obtained from UniProt using keyword-based exclusion of toxin- and antimicrobial-related entries. The combined dataset was then filtered for inconsistent annotations and clustered at 90% AAI with CD-HIT. We used the released benchmark, comprising 2,138 toxic and 5,375 non-toxic peptides.

For bacteriocin prediction, we used the benchmark dataset from BaPreS (Akhter and Miller, 2023), comprising experimentally validated bacteriocin sequences from the BAGEL and BACTIBASE databases, along with non-bacteriocin sequences from the RMSCNN dataset; redundancy was removed using CD-HIT at 90% sequence identity. The dataset was restricted to sequences no longer than 100 amino acids to align with the definition of small proteins, yielding 242 sequences (229 bacteriocin and 13 non-bacteriocin) for evaluation.

For anti-CRISPR (Acr), we used the benchmark dataset released with PaCRISPR (Wang et al., 2020), which includes experimentally characterized Acr proteins sourced from anti-CRISPRdb, together with non-Acr negative sequences derived from Acr-containing phages and bacterial mobile genetic elements. After length filtering, 294 sequences (47 Acr and 247 non-Acr) were retained.

For quorum-sensing peptide prediction, we used the benchmark dataset employed by Rajput et al. (2015), comprising 220 experimentally verified quorum-sensing peptides sourced from the Quorumpeps database and 220 non-QSP negative sequences, totalling 440 balanced sequences.

For cell-penetrating peptide prediction, we used the benchmark dataset established in the pLM4CPPs framework (Kumar et al., 2025), integrating experimentally validated CPPs from CPPsite2.0, C2Pred, CellPPD, MLCPP 2.0, and KELM-CPPpred. After redundancy removal, the dataset comprises 1,399 CPP and 4,080 non-CPP.

### 2.5 Frozen-encoder downstream evaluation

For each pre-trained encoder (including TinyProteinTransformer and baselines), we froze the encoder parameters and extracted fixed-dimensional sequence embeddings. For TinyProteinTransformer, embeddings were obtained from the learned attention pooling layer (640 dimensions). For ESM-series models and ProtBERT, we used mean pooling over the last layer representations, since they do not provide attention pooling.

A single linear classification head (nn. Linear(embedding_dim, 2)) was trained from scratch for each encoder. The AdamW optimizer (learning rate = 1e-3, weight decay = 0.01) was used with cross-entropy loss. Training ran for exactly 5 epochs without early stopping or learning-rate scheduling, with a batch size of 64. To ensure reliable and unbiased performance estimates, we employed 5-fold stratified cross-validation (StratifiedKFold from scikit-learn, shuffle = True, random_state = 42), preserving class proportions in each fold. In each fold, 80% of the data were used to train the linear head, and 20% were used for validation.

Sequence representations were pre-computed in batches using frozen encoders to avoid redundant forward passes. Evaluation metrics included accuracy (ACC), macro F1-score, and area under the ROC curve (AUC), computed using scikit-learn functions. Metrics were averaged across the 5 folds, with standard deviations reported as ± values. All experiments fixed a global random seed of 42 (via the seed_everything function) to ensure full reproducibility across random splits, model initialization, and data shuffling.

### 2.6 Comparison with non-pretrained prediction methods

To contextualize TPT’s pretrain-then-probe performance against non-pretrained supervised baselines, we further evaluated three sequence classifiers trained end-to-end from random initialization on the AMP benchmarks: AMP Scanner v2 (Veltri et al., 2018), comprising an embedding layer, a single 1D convolutional layer (64 filters, kernel size 16), a max-pooling layer, and an LSTM layer (100 units); AMPlify (Li C. et al., 2022), comprising a bidirectional LSTM (512 units per direction) followed by 32-head scaled dot-product attention and a context attention pooling layer; and a Fast-MCWS Transformer (Mahala et al., 2025), comprising a convolutional front-end with average pooling followed by three parallel windowed self-attention blocks at 25, 50, and 100% of the pooled sequence length. All three models were implemented in PyTorch and trained under identical conditions: AdamW optimizer, learning rate 1 × 10^−3^, batch size 32–64, maximum 200 epochs with early stopping monitored on validation accuracy (patience = 10), and the same 5-fold stratified splits (StratifiedKFold, random_state = 42) as the frozen-encoder evaluation. No pretrained weights were loaded. AMP Scanner v2 and AMPlify used one-hot or integer-encoded amino acid sequences as input; Fast-MCWS used a 4-mer dictionary tokenization scheme built from the downstream dataset sequences. This setup reflects each method’s intended supervised use case and enables direct comparison of end-to-end training against TPT’s pretrain-then-probe paradigm.

### 2.7 Ablation studies

To evaluate the contribution of key architectural components and training objectives, we constructed several TPT variants by removing or replacing one component at a time. TPT-NoCL was trained using only the masked language modeling objective, with the InfoNCE contrastive loss removed. TPT-MeanPool replaced the learned attention-pooling module with mean pooling over all non-padding residue positions. TPT-NoCNN removed the parallel multi-scale convolutional branch and passed token embeddings directly into the Transformer encoder. In addition, TPT-Gated was evaluated as a gated-attention variant to examine the effect of head-specific sigmoid gating.

All ablation variants were evaluated using the identical downstream protocol as the full model: frozen encoder embeddings extracted via attention pooling (or mean pooling for TPT-MeanPool), a single linear classification head trained for 5 epochs (AdamW, lr = 1 × 10^−3^, weight decay = 0.01), and 5-fold stratified cross-validation across all four benchmarks. Results are reported as mean ± standard deviation of ACC, macro F1, and AUC across folds.

### 2.8 Representation visualization

To qualitatively compare how different pre-trained encoders construct peptide representations, we performed a two-dimensional t-SNE visualization of the toxicity dataset (Van der Maaten and Hinton, 2008). For each sequence, a fixed-length embedding is obtained using the frozen encoder. The t-SNE (n_components = 2, perplexity = 30, learning_rate = 200, random_state = 42) implemented in scikit-learn was used to reduce the dimensionality of each model independently.

## 3 Results

### 3.1 Pretraining loss dynamics

TPT was pretrained on microbial small-protein sequences from the GMSC10.90 catalog, using a joint objective that combined masked language modeling (MLM) with a contrastive loss. The MLM term encouraged the model to recover masked residues from the local sequence context. Meanwhile, the contrastive term aligned augmented views of the same sequence in the embedding space. Figure 2 shows the two loss curves over 50,000 training steps. Both terms decreased rapidly during early training and then converged to stable plateaus. The MLM loss settled at approximately 1.8, with only minor late-stage fluctuations. The contrastive loss fell to consistently low values, and we observed no signs of representation collapse. Together, these dynamics indicate that the two objectives were optimized in a stable manner and provided a well-behaved starting point for downstream evaluation.

### 3.2 Overall performance across six downstream tasks

We systematically evaluated our TPT and TPT_gated models against three pretrained baselines (ESM2-35 M, ESM2-150 M, and ProtBERT). The six tasks covered functionally distinct classes of microbial peptides and proteins: antimicrobial peptides (AMP), toxic peptides (TOX), bacteriocins (BCN), anti-CRISPR proteins (Acr), quorum-sensing peptides (QSP), and cell-penetrating peptides (CPP), assessed using 5-fold stratified cross-validation. All encoders were frozen and evaluated with a linear classification head, so that performance reflected the quality of pretrained representations. Accuracy, F1, and AUC are reported in Supplementary Table S7.

Because ESM2 and ProtBERT were evaluated using mean-pooled residue embeddings, we also included TPT-MeanPooling in Supplementary Table S7 as a common mean-pooling comparison. This variant replaced the learned attention-pooling module with mean pooling while keeping the remaining TPT encoder unchanged. TPT-MeanPooling remained competitive with the baseline PLMs across the evaluated tasks, suggesting that TPT performance was not solely driven by the attention-pooling module.

On AMP and TOX benchmarks, the TPT showed competitive performance compared with the pretrained baseline models. For AMP, TPT obtained the highest mean AUC (0.930 ± 0.008), but this did not differ significantly from ESM2-150 M (0.928 ± 0.004; *p* = 0.68). A similar pattern held for TOX, where TPT had the highest mean AUC (0.930 ± 0.006), followed closely by ESM2-150 M (0.925 ± 0.009; *p* = 0.12).

On BCN, all models reached accuracy between 0.94 and 0.97. ESM2-150 M obtained the highest mean AUC (0.955 ± 0.040) and TPT_gated the second-highest (0.947 ± 0.045), but the two were again statistically indistinguishable (*p* = 0.77). Notably, TPT_gated achieved the best F1 (0.983 ± 0.015). The wide confidence intervals on this task reflect its small, unbalanced benchmark and limited resolution between comparisons.

On QSP and CPP, TPT also showed strong performance. For QSP, TPT achieved the best overall results among all compared models, with the highest ACC (0.868 ± 0.027), F1 score (0.872 ± 0.031), and AUC (0.923 ± 0.021). For CPP, TPT again achieved the highest overall performance, with an ACC of 0.901 ± 0.008, F1 score of 0.793 ± 0.022, and AUC of 0.938 ± 0.009. TPT_gated showed comparable performance on CPP, with an AUC of 0.937 ± 0.009, indicating that both TPT variants retained effective on these peptide-related tasks. Across the six tasks, the two TPT variants showed complementary behavior: the base TPT model performed strongly on AMP, TOX, QSP, and CPP, whereas TPT_gated performed particularly well on BCN.

The Acr task revealed a clear and significant separation between the two model families. The three pretrained baselines collapsed to majority-class prediction, with an F1 of 0.000 and an AUC at or below chance (ESM2-35 M 0.463, ESM2-150 M 0.428, ProtBERT 0.322). In contrast, TPT and TPT_gated retained predictive signal, achieving AUCs of 0.696 ± 0.071 and 0.692 ± 0.098, respectively, with non-trivial F1 scores. The advantage of TPT over all baselines was consistent across all five folds and statistically significant; for example, *p* = 0.015 against ESM2-150 M and *p* = 0.0004 against ProtBERT. The same pattern was evident in the embedding geometry. On Acr, all three baselines yielded negative silhouette coefficients (−0.034 to −0.050), indicating that samples lay closer to the opposite class on average, whereas TPT retained a positive coefficient. Silhouette values for the evaluated tasks are reported in Supplementary Table S3. One possible explanation for this robustness is the contrastive component of pretraining. By encouraging distinct sequences to occupy more separable regions in the embedding space, contrastive learning may help preserve minority-class signals that are difficult to capture using MLM-only objectives under strongly imbalanced settings. This interpretation is further supported by the ablation results (Supplementary Table S8).

To further evaluate TPT against task-specific SOTA methods, we compared it with two representative state-of-the-art AMP prediction models, AMPlify and AMPScannerV2, using the same AMP benchmark dataset and evaluation protocol. As shown in Table 1, TPT achieved the best overall performance among the compared methods. This additional comparison further supports the effectiveness of TPT for AMP prediction.

**Table 1:** Comparison of TPT with representative non-pretrained methods on the AMP prediction task.

Model	Training setting	ACC	F1	AUC
TPT-FullFinetune	Full fine-tuning	0.9161 ± 0.0084	0.9157 ± 0.0079	0.9733 ± 0.0038
TPT-Gated-FullFinetune	Full fine-tuning	0.9099 ± 0.0068	0.9102 ± 0.0075	0.9692 ± 0.0061
AMPScannerV2	Trained from scratch	0.9035 ± 0.0022	0.9011 ± 0.0024	0.9555 ± 0.0020
AMPlify	Trained from scratch	0.8766 ± 0.0241	0.8752 ± 0.0238	0.9449 ± 0.0174
FastMCWS-FromScratch	Trained from scratch	0.8663 ± 0.0100	0.8651 ± 0.0087	0.9406 ± 0.0045

All models were retrained and evaluated on the same AMP benchmark dataset using the same data splits and evaluation protocol. AMPlify and AMPScannerV2 were included as representative AMP-specific prediction methods, and FastMCWS-FromScratch was included as an MCWS-based comparison. ACC, F1 score, and AUC are reported as mean ± standard deviation across five folds where available.

### 3.3 Contribution of each component

To assess the contribution of key model components, we compared the full TPT model with ablation variants removing contrastive learning, replacing attention pooling with mean pooling, removing the multi-scale CNN branch, or using gated attention (Supplementary Table S8). Removing attention pooling or the CNN branch led to moderate performance reductions across most tasks, as reflected by the AMP AUC decreasing from 0.929 to 0.916 and 0.917, respectively. In contrast, removing the contrastive objective caused the largest performance drop, with AUC decreasing from 0.929 to 0.869 on AMP (*p* = 0.0001), from 0.955 to 0.716 on BCN (*p* = 0.007), and from 0.733 to 0.528 on Acr (*p* = 0.024). These results suggest that local motif extraction and adaptive pooling contribute to TPT performance, while contrastive learning provides a particularly important sequence-level signal for learning discriminative representations, especially in imbalanced or low-resource tasks.

### 3.4 Impact of hidden dimension

To determine the optimal representational capacity for TPT, we evaluated the effect of the Transformer’s hidden dimension size. We trained variants of the model with hidden dimensions of 320, 480, 640, and 800, keeping all other hyperparameters constant, and assessed their downstream classification performance (Supplementary Table S9).

The results revealed a clear performance peak at a hidden dimension of 480 and 640. On the challenging and highly imbalanced Acr task, increasing the dimension from 320 to 640 substantially improved the mean AUC from 0.561 ± 0.123 to a peak of 0.733 ± 0.040. However, further expanding the capacity to 800 resulted in a performance drop (AUC 0.631 ± 0.094), suggesting the onset of overfitting on the rare minority class. A similar pattern emerged on the AMP benchmark, where the 640-dimensional model achieved the highest mean AUC of 0.930 ± 0.003.

For the toxicity prediction (TOX) task, performance was remarkably stable across all tested capacities, with mean AUCs consistently plateauing between 0.925 and 0.928. This indicates that the task is relatively insensitive to model scaling once a baseline representation capacity is reached. While a smaller dimension (480) yielded a higher mean AUC on the small bacteriocin (BCN) dataset, the 640-dimensional variant maintained an exceptionally high F1 score (0.964 ± 0.020) well within the margin of the dataset’s high variance. Consequently, a hidden dimension of 640 was established as the default configuration for TPT. It possesses sufficient capacity to resolve complex sequence features without over-parameterizing and destabilizing the learning dynamics on limited microbial datasets.

### 3.5 Model size, inference cost, and accuracy

**Figure 3:**
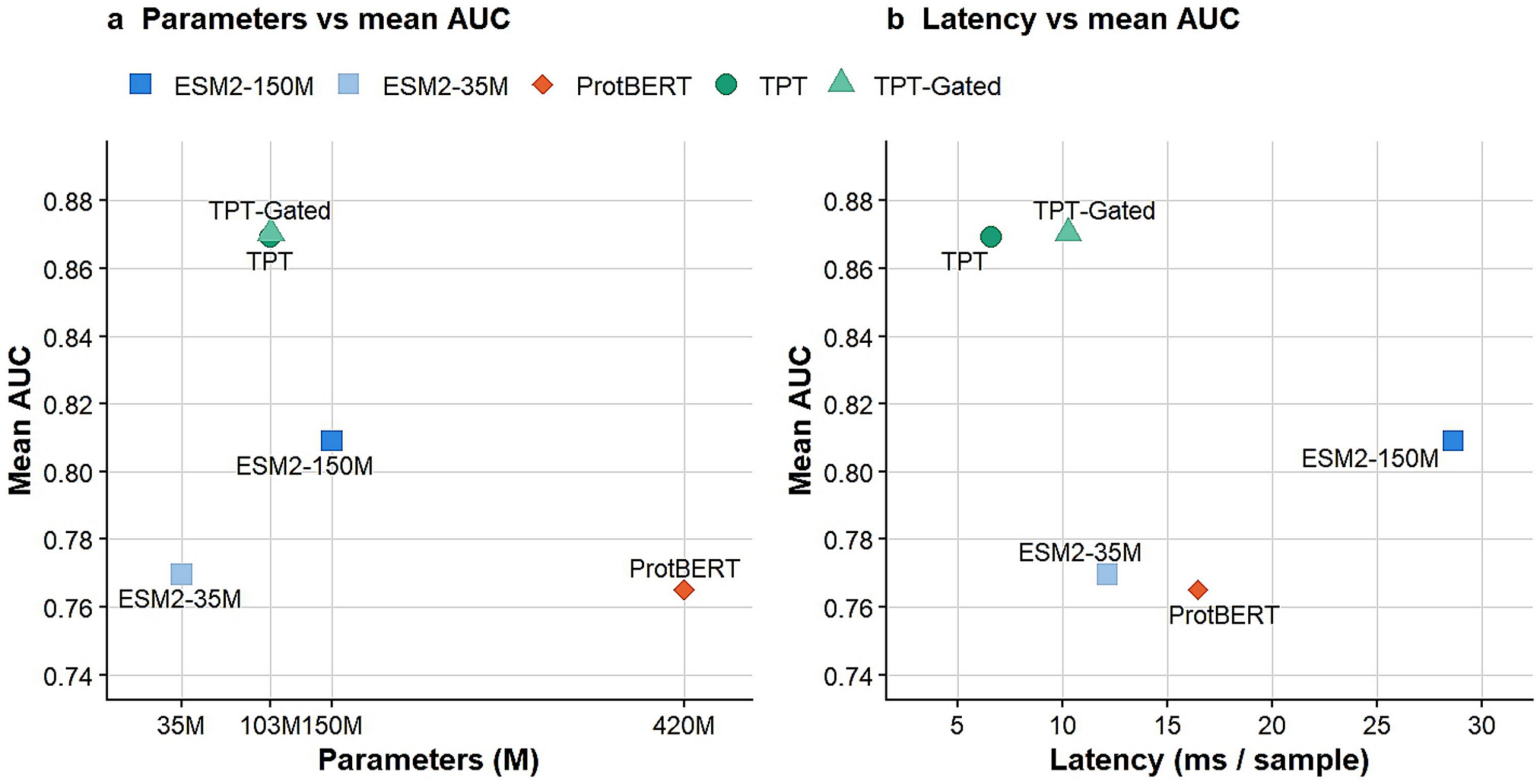
Parameter and inference efficiency of TPT. Model performance was evaluated on the six downstream prediction task. **(a)** Mean AUC plotted against model parameter size. **(b)** Mean AUC plotted against inference latency. Latency is reported as milliseconds per sample. All encoders were evaluated on the same NVIDIA RTX 5090 GPU.

Having observed that TPT achieved competitive performance across the evaluated benchmarks, we next examined its model size and inference cost (Figure 3). TPT contains 103 M parameters, fewer than ESM2-150 M (150 M) and ProtBERT (420 M), but more than ESM2-35 M (35 M). Among the baseline models, ESM2-150 M generally performed better than ESM2-35 M, indicating that model scale can still be beneficial. However, under the current evaluation setting, TPT achieved competitive performance while maintaining a smaller parameter count than ESM2-150 M and ProtBERT (Figure 3a).

We further compared inference latency across models (Figure 3b; Supplementary Table S5). TPT encoded a single sequence in approximately 6.6 ms on one NVIDIA RTX 5090 GPU, compared with approximately 12 ms for ESM2-35 M, 16.5 ms for ProtBERT, and 28.6 ms for ESM2-150 M. This lower inference cost may be related to the compact CNN–Transformer architecture and lightweight sequence-processing pipeline used in TPT. Taken together, these results suggest that, within the evaluated benchmarks, TPT provides a favorable balance between predictive performance, model size, and inference efficiency, rather than indicating that model scale is unimportant in general.

### 3.6 Representation, geometric and class structure

**Figure 4:**
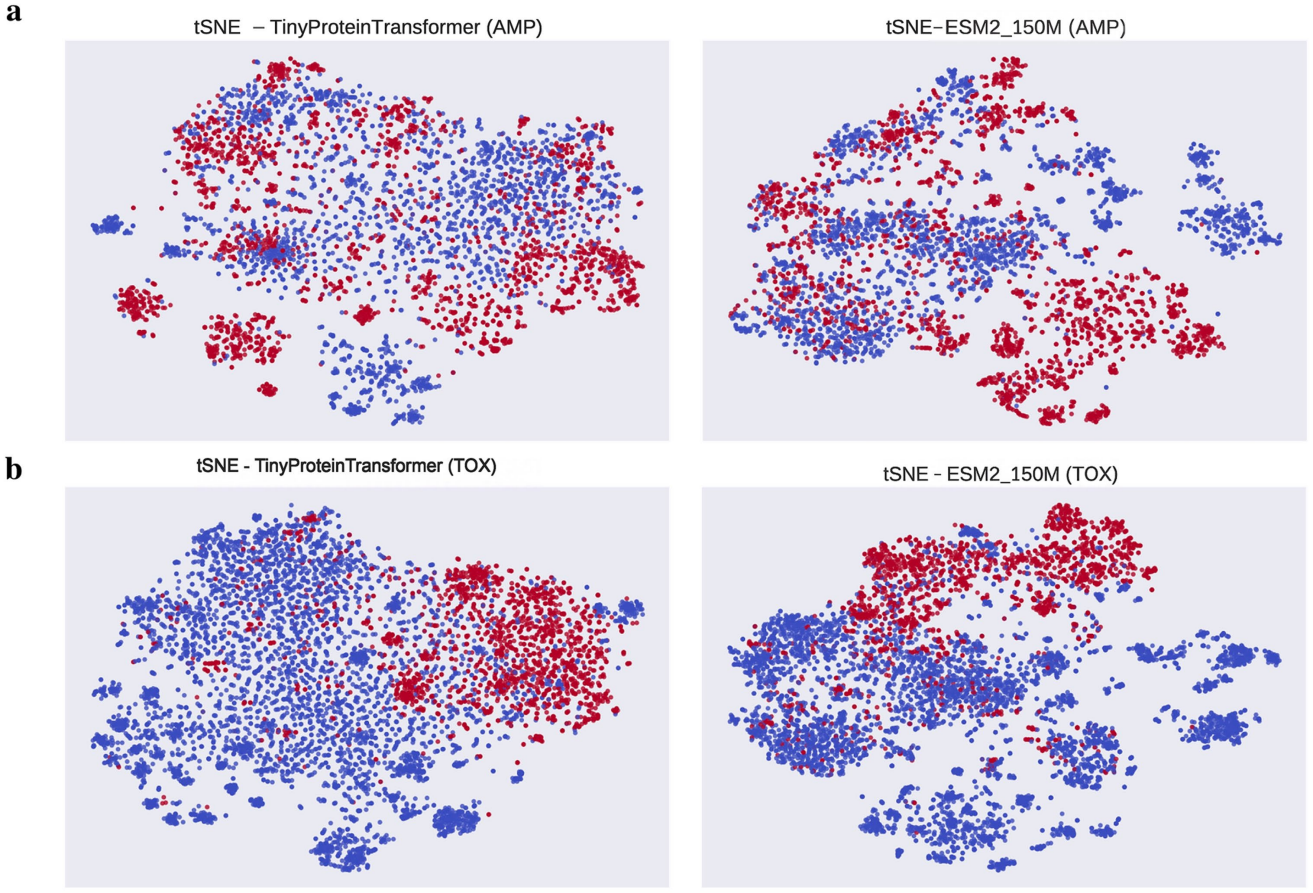
t-SNE visualization of peptide embeddings across different downstream tasks. Two-dimensional t-SNE projections of embeddings were generated using TinyProteinTransformer and ESM2-150M. **(a)** t-SNE visualization for the AMP task. **(b)** t-SNE visualization for the TOX task. Red and blue dots indicate the two class labels in each task.

To further examine the representation structure learned by different encoders, we projected frozen encoder embeddings into two dimensions using t-SNE (Figure 4). The complete t-SNE projections for the remaining model–task combinations are provided in Supplementary Figure S1. On the toxicity task, TPT showed a relatively clear visual separation between toxic and non-toxic peptides, whereas the baseline models displayed more fragmented projections with greater class overlap. On the AMP task, all models showed noticeable overlap between AMP and non-AMP sequences, indicating that two-dimensional t-SNE projections alone may not fully capture the discriminative structure of the embedding space. To provide a quantitative complement to the t-SNE visualization, we further calculated silhouette coefficients for each model across downstream tasks (Supplementary Table S3). The silhouette coefficients did not always parallel the linear-probe AUC results. For AMP and TOX, TPT achieved the highest AUCs despite having lower silhouette coefficients than some baseline models. In contrast, on the Acr task, TPT was the only model with a positive silhouette coefficient, whereas all baseline models showed negative values. These results suggest that t-SNE and silhouette analyses provide complementary information about embedding geometry and should be interpreted together with downstream classification metrics.

## 4 Discussion

The results suggest that modeling of microbial small proteins may benefit from architectural approaches that differ from those used in large protein language models (PLMs). Most existing PLMs are trained primarily on long, evolutionarily conserved, and domain-rich proteins (Elnaggar et al., 2021; Lin et al., 2023). In contrast, smORFs are characterized by rapid evolutionary turnover, relatively sparse domain organization, and frequent reliance on short functional motifs (Durrant and Bhatt, 2021; Miravet-Verde et al., 2019; Storz et al., 2014). These features may limit the effectiveness of architectures designed mainly to capture long-range dependencies and deep evolutionary signals.

To address this mismatch, we developed TinyProteinTransformer (TPT), which combines convolution-based motif extraction with lightweight Transformer-based contextual modeling. This design is motivated by previous studies suggesting that CNNs can effectively detect local biochemical patterns relevant to peptide function (Veltri et al., 2018), while Transformer layers provide a flexible mechanism for modeling broader sequence context. The combination of these two inductive biases offers a plausible explanation for the favorable efficiency–performance trade-off observed for TPT. Furthermore, contrastive learning, employed in this work largely as a regularizer, is known to induce functional clustering even without homology (Lu et al., 2020), which is consistent with the coherent geometric structure revealed in the TPT embeddings. We also note that alternative approaches have been proposed to introduce locality into protein sequence modeling. For example, MCWS-Transformers employ multi-context-window self-attention to capture local sequence context, while subsequent CNN + MCWS architectures combine convolutional operations with localized attention mechanisms for efficient protein function prediction (Ranjan et al., 2023; Mahala et al., 2025). These studies further highlight the importance of explicitly modeling local sequence patterns in protein representation learning.

This work also has broader implications for protein representation learning in microbial genomics. As GMSC and other large-scale metagenomic resources continue to expand the number of reported microbial peptides, the domain-adapted encoder may become increasingly important. In high-throughput microbiome studies, where millions of candidate smORFs may need to be screened (Steinegger and Söding, 2018), parameter-efficient models provide a practical alternative to multi-billion-parameter PLMs. The performance of TPT suggests that the effective representation of smORFs may depend not only on model scale, but also on architectural priors tailored to the sequence and biochemical characteristics of short microbial proteins.

The ablation experiments further support the contribution of the main TPT components. Removing attention pooling or the CNN module caused moderate performance reductions, suggesting that adaptive residue-level aggregation and local motif extraction both contribute to the learned representations. In contrast, removing the contrastive objective led to the most consistent decline across the four downstream tasks, particularly on the imbalanced Acr task, where AUC approached the chance level. These findings suggest that contrastive learning provides an important sequence-level discriminative signal that complements the residue-level MLM.

The effect of gated attention was also task-dependent across the four downstream benchmarks. Compared with the base TPT model, TPT_gated did not provide consistent improvements on AMP, TOX, or Acr, but showed better performance on the BCN task. This suggests that gated attention is not universally beneficial, and its utility may depend on the type of sequence signal required by each task. One possible explanation is that gating may be beneficial when functional information is concentrated in a limited set of informative residues or local motifs (Rathore et al., 2024), as may occur in some bacteriocin-related sequences. In contrast, for tasks such as AMP prediction, where activity may depend more strongly on distributed physicochemical properties such as net charge and hydrophobicity (Hancock and Sahl, 2006; Wimley, 2010), gating may attenuate globally informative signals. These findings indicate that gated attention should be considered a task-dependent module rather than a generally superior replacement for standard attention.

Silhouette coefficients and AUC scores conveyed complementary but distinct aspects of representation geometry. On AMP and TOX tasks, TPT achieved the highest AUC values while yielding lower silhouette coefficients than the baselines (Supplementary Table S3). This reflects a known dissociation between cluster compactness and linear separability: the contrastive objective may produce embeddings well-organized for linear discrimination without forming compact globular clusters. On the Acr task, all three baselines yielded negative silhouette coefficients, indicating that their embeddings placed Acr sequences closer to the opposite class on average, consistent with their collapse under linear probing. TPT retained the only positive silhouette coefficient on this task, aligning with its substantially higher AUC.

Several limitations of this study should be acknowledged. (1) Functional annotations for smORFs remain relatively limited, constraining the size and diversity of available benchmark datasets. (2) Our downstream evaluation was conducted using a frozen-encoder linear-probing framework to enable fair comparisons across models. Although this strategy provides a standardized evaluation setting, it may not fully reflect the achievable performance under end-to-end fine-tuning. (3) While TPT integrates both local and global sequence information, it does not currently incorporate structural supervision, which has recently shown favorable effects in large-scale PLMs (Hartout et al., 2025; Lin et al., 2023; Wu et al., 2024; Zhang et al., 2024). (4) Potential sequence overlap between the GMSC10.90 pretraining corpus and downstream benchmark datasets should also be considered. Although exact sequence overlap was limited overall, near-identity overlap showed dataset-dependent variation and was relatively higher in several small benchmarks, such as Acr and BCN (Supplementary Table S1). These overlaps are partly expected because smORF-encoded peptides are short and the GMSC corpus is extremely large, making near-identical short peptide sequences difficult to completely avoid. Because TPT was pretrained in a self-supervised manner without downstream task labels, this does not represent supervised label leakage. Nevertheless, sequence-level homology between pretraining and downstream datasets may still influence performance estimates. Future work should therefore explore the integration of geometric or structure-aware training objectives, as well as broader evaluation across additional classes of smORF-associated functions, including regulatory, signaling, and membrane-associated micropeptides.

Taken together, our findings suggest that effective modeling of microbial smORFs depends not only on model scale, but also on inductive biases tailored to the sequence characteristics of short, rapidly evolving proteins. By integrating local motif extraction with Transformer-based contextual modeling, TPT provides a compact and computationally efficient framework for learning microbial peptide representations. While further validation across a broader range of smORF-associated functions is needed, this work highlights the potential of specialized protein encoders as scalable tools for peptide analysis in microbial genomics.

## 5 Conclusion

In conclusion, this study demonstrates that accurate modeling of microbial smORFs requires architectures informed by their distinct biological properties, rather than directly transferring designs optimized for longer proteins. Protein sequence modeling stands to benefit from domain- and length-specialized architectures rather than relying solely on ever-larger general-purpose models. While training universal PLMs incurs high computational cost and inference latency, compact, domain-adapted encoders such as TPT offer a more practical and scalable solution for large-scale peptide analysis. By combining local motif extraction with robust context modeling, TPT provides an efficient, biologically motivated framework for advancing small protein research in microbial genomics.

## Data Availability

The original contributions presented in the study are included in the article/Supplementary material. The code and data used to reproduce the analyses are available at https://github.com/F4NG66/TPT. Further inquiries can be directed to the corresponding author.
