# Supplementary material for: TPT: a compact CNN-transformer encoder for efficient microbial small protein modeling
Source: Front Microbiol. 2026 Aug 3;17:1839420. doi: 10.3389/fmicb.2026.1839420 (PMC13478124; doi:10.3389/fmicb.2026.1839420)
Supplement: Supplementary file 1 [file Table_1.DOCX]

**Supplementary Table S1. Sequence overlap analysis between the GMSC pretraining corpus and downstream benchmark datasets.**

| **Dataset** | **Total sequences** | **Exact hits in GMSC** | **Exact overlap (%)** | **Near-identity hits in GMSC** | **Near-identity overlap (%)** |
| --- | --- | --- | --- | --- | --- |
| **AMP** | 8,346 | 194 | 2.32 | 820 | 9.83 |
| **TOX** | 7,513 | 403 | 5.36 | 1,370 | 18.24 |
| **Acr** | 294 | 35 | 11.9 | 188 | 63.95 |
| **BCN** | 242 | 45 | 18.6 | 110 | 45.45 |
| **QSP** | 440 | 14 | 3.18 | 25 | 5.68 |
| **CPP** | 5,479 | 0 | 0 | 9 | 0.16 |
| **Total** | 22,314 | 691 | 3.1 | 2,522 | 11.3 |

Exact hits indicate identical sequence matches between downstream benchmark sequences and the GMSC10.90 pretraining corpus. Near-identity hits were identified using MMseqs2 easy-search with thresholds of ≥90% sequence identity and ≥80% coverage
