# Supplementary material for: TPT: a compact CNN-transformer encoder for efficient microbial small protein modeling
Source: Front Microbiol. 2026 Aug 3;17:1839420. doi: 10.3389/fmicb.2026.1839420 (PMC13478124; doi:10.3389/fmicb.2026.1839420)
Supplement: Supplementary file 2 [file Table_2.DOCX]

**Supplementary Table S2. TinyProteinTransformer architectural and training hyperparameters**

| Hyperparameter | Value | Rationale / note |
| --- | --- | --- |
| *Input & tokenization* | | |
| Max sequence length | 128 | Covers >99% of GMSC smORF lengths |
| Vocabulary size | 25 | 20 canonical AAs + B, X, Z + <MASK> + <PAD> |
| Embedding dimension (dmodel) | 640 | Balances representational capacity and pretraining compute |
| Positional embedding | Learned | Trainable lookup; not sinusoidal |
| *Multi-scale CNN module* | | |
| Kernel sizes | 3, 5, 7, 9 | Spans typical functional motif lengths in smORFs |
| Channels per branch | 256 | 1,024 total after concatenation, projected to 640 |
| Activation | ReLU | — |
| *Transformer encoder* | | |
| Number of layers | 20 | — |
| Attention heads | 10 | 64 dimensions per head |
| Feed-forward dimension | 2,560 | 4 × dmodel |
| Dropout | 0.05 | Conservative; large pretraining corpus reduces overfitting risk |
| Layer normalization | Pre-LN | norm_first=True; improves training stability |
| *Attention pooling* | | |
| Projection | 640 → scalar | Per-position importance score; softmax-normalized |
| Output embedding | 640 | Weighted average over L residue positions |
| *Gated attention (TPT-Gated variant only)* | | |
| Gate function | σ(H·Wg) | Wg ∈ ℝ^(d×H); head-specific sigmoid gate |
| Extra parameters | None | Reuses existing attention weight matrices |
| *Masked language modeling* | | |
| Overall masking rate | 15% | Following BERT (Devlin et al., 2019) |
| Span masking probability | 30% | Targets contiguous motif regions; span length 2–5 residues |
| *Contrastive learning* | | |
| Temperature (τ) | 0.1 | Standard InfoNCE practice (van den Oord et al., 2018) |
| Loss weight (λ) | 0.05 | MLM dominant; contrastive loss as embedding regularizer |
| Augmentation 1 — random deletion | p = 0.30 | Length 2–5 residues; mimics natural sequence truncations |
| Augmentation 2 — conservative substitution | p = 0.15 | Per position; within physicochemical groups |
| *Pretraining optimization* | | |
| Optimizer | AdamW | Loshchilov & Hutter, 2019 |
| Learning rate | 2×10⁻⁵ | Constant; no LR scheduler |
| Weight decay | 0.01 | AdamW default |
| Batch size | 200 | — |
| Epochs | 3 | Sufficient for stable loss plateau on GMSC scale |
| Precision | Mixed (AMP) | torch.cuda.amp; autocast + GradScaler |
| Hardware | 2× NVIDIA RTX 5090 | DataParallel; gradient sync per step |
| Random seed | 42 | All pretraining and downstream experiments |
| *Downstream linear probing* | | |
| Classification head | Linear(640, 2) | No hidden layers; encoder frozen |
| Optimizer | AdamW | lr = 1×10⁻³, weight decay = 0.01 |
| Epochs | 5 | No early stopping or LR scheduling |
| Batch size | 64 | — |
| Cross-validation | 5-fold stratified | random_state = 42; 80/20 train/val per fold |
| Evaluation metrics | ACC, macro F1, AUC | Mean ± SD across 5 folds |
