# Supplementary material for: TPT: a compact CNN-transformer encoder for efficient microbial small protein modeling
Source: Front Microbiol. 2026 Aug 3;17:1839420. doi: 10.3389/fmicb.2026.1839420 (PMC13478124; doi:10.3389/fmicb.2026.1839420)
Supplement: Supplementary file 3 [file Table_3.DOCX]

**Supplementary Table S3. Silhouette scores of peptide embeddings across downstream tasks.**

| **Task** | **TPT** | **ESM-2 35M** | **ESM-2 150M** | **ProtBERT** |
| --- | --- | --- | --- | --- |
| **AMP** | 0.0195 | 0.053 | 0.0536 | 0.0911 |
| **TOX** | 0.0425 | 0.0616 | 0.0575 | 0.0677 |
| **Acr** | 0.0024 | -0.0399 | -0.0337 | -0.0495 |
| **BCN** | 0.006 | 0.1094 | 0.0956 | 0.1398 |
