# Supplementary material for: TPT: a compact CNN-transformer encoder for efficient microbial small protein modeling
Source: Front Microbiol. 2026 Aug 3;17:1839420. doi: 10.3389/fmicb.2026.1839420 (PMC13478124; doi:10.3389/fmicb.2026.1839420)
Supplement: Supplementary file 4 [file Table_5.DOCX]

**Supplementary Table S5. Inference latency comparison of TPT variants and baseline protein language models.**

| **Category** | **Model** | **Latency_ms (meanÂ±sd over 6 datasets)** | **Relative_latency** |
| --- | --- | --- | --- |
| **Ours** | **TPT (Attention) (ref)** | 6.60 ± 0.19 | 1 |
| **Ours** | **TPT (Gated)** | 10.25 ± 0.42 | 1.55 |
| **Ablation** | **w/o Attention** | 6.58 ± 0.30 | 1 |
| **Ablation** | **w/o CNN** | 6.01 ± 0.25 | 0.91 |
| **Ablation** | **w/o Loss** | 6.39 ± 0.11 | 0.97 |
| **Baseline** | **ESM-2 (35M)** | 12.12 ± 0.21 | 1.84 |
| **Baseline** | **ESM-2 (150M)** | 28.63 ± 0.85 | 4.34 |
| **Baseline** | **ProtBERT** | 16.45 ± 0.38 | 2.49 |
