# Supplementary material for: TPT: a compact CNN-transformer encoder for efficient microbial small protein modeling
Source: Front Microbiol. 2026 Aug 3;17:1839420. doi: 10.3389/fmicb.2026.1839420 (PMC13478124; doi:10.3389/fmicb.2026.1839420)
Supplement: Supplementary file 5 [file Table_6.DOCX]

**Supplementary Figure S1.** t-SNE visualization of frozen encoder embeddings across all downstream tasks and models.
